# Dietary regulation of hypodermal polyploidization in *C. elegans*

**DOI:** 10.1186/1471-213X-8-28

**Published:** 2008-03-12

**Authors:** Luke S Tain, Encarnación Lozano, Alberto G Sáez, Armand M Leroi

**Affiliations:** 1 Department of Biomedical Sciences, University of Sheffield, Sheffield, S10 2TN, UK; 2 Department of Biological Sciences, Silwood Park Campus, Imperial College London, Ascot, Berkshire SL5 7PY, U.K; 3 Museo Nacional de Ciencias Naturales (CSIC), Dept. Biodiversidad y Biología Evolutiva, C/José Gutiérrez Abascal 2, 28006 Madrid, Spain

## Abstract

**Background:**

Dietary restriction (DR) results in increased longevity, reduced fecundity and reduced growth in many organisms. Though many studies have examined the effects of DR on longevity and fecundity, few have investigated the effects on growth.

**Results:**

Here we use *Caenorhabditis elegans *to determine the mechanisms that regulate growth under DR. We show that rather than a reduction in cell number, decreased growth in wild type *C. elegans *under DR is correlated with lower levels of hypodermal polyploidization. We also show that mutants lacking wild type sensory ciliated neurons are small, exhibit hypo-polyploidization and more importantly, when grown under DR, reduce their levels of endoreduplication to a lesser extent than wild type, suggesting that these neurons are required for the regulation of hypodermal polyploidization in response to DR. Similarly, we also show that the cGMP-dependent protein kinase EGL-4 and the SMA/MAB signalling pathway regulate polyploidization under DR.

**Conclusion:**

We show *C. elegans *is capable of actively responding to food levels to regulate adult ploidy. We suggest this response is dependent on the SMA/MAB signalling pathway.

## Background

Many animals change their life-history, size or shape in response to the environment; a phenomenon known as phenotypic plasticity [1,2]. One environmental factor that exerts great influence over the development and life history of an organism is that of nutrition, or 'dietary restriction' [3-8]. Studies in a variety of taxa have shown that restricting the nutrition of juveniles or adults reduces growth and fecundity, while increasing longevity [9-11].

Over the last decade the underlying cellular mechanisms that regulate the effect of DR on growth have been explored more extensively [12]. In metazoans, it appears that much of an organism's ability to respond to DR is determined by insulin-like signalling. For example, overexpression of Insulin-like Growth Factor Binding Protein-1 (IGFBP-1) is known to cause retardation of bone growth [13] and is found in DR rats at three times the normal level [14]. *Drosophila *and mice lacking components of the Insulin-like signalling pathway have greatly reduced body [15-19]. This reduction in size is due to a combination of reduced cell number and cell size [18,19]. In contrast, insulin-associated pathways in *C. elegans *are known to determine fat storage, diapause, and longevity, but their effect on body size is less evident [20-25]. However, genetic mechanisms of body size determination in *C. elegans *are known to involve DBL-1 signalling (TGF-β ligand homologous to Drosophila's *Dpp *and vertebrate's BMP). DBL-1 regulates normal growth in *C. elegans *through the SMA/MAB pathway [26], along with downstream components such as LON-1 [27,28]. It seems to us a reasonable hypothesis that the DBL-1 signalling may be involved in the DR response. Moreover, this relationship may extend to sensory-based regulation of growth. Mutant strains lacking properly formed and functional sensory ciliated neurons, such as the *che *mutants (cilia extension defects), together with downstream cGMP-dependent protein kinase EGL-4, exhibit alterations not only in longevity but also in body size [29-31].

In this study we investigate whether *C. elegans *undergoes a programmed regulation of growth in response to DR. First, we characterized life history responses, of wild type *C. elegans*, to DR, determining longevity, fecundity and body size. Second, we determined the role of the sensory system in growth regulation in response to DR. Thirdly, we examined the role of TGF-β signalling in DR mediated growth responses and determine how this relates to the sensory system.

## Results

### Dietary restriction in *C. elegans *reduces body size, hypodermal ploidy and fecundity but increases longevity

**Figure 1:**
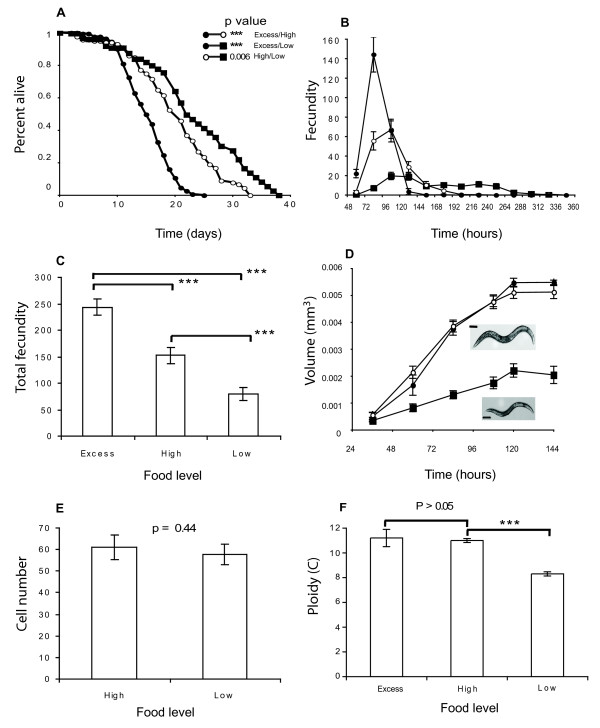
**The effects of dietary restriction on *C. elegans *life history traits**. (A) Kaplan-Maier survival curves showing the longevity of *C. elegans *under excess (closed circles), high (open circles) and low food (closed squares) environments (see Material and Methods). Significance is shown for excess, high, and low food, from Log Rank tests *n*/censored individuals 175/22, 204/110 and 238/189 respectively. (B) Daily fecundity of *C. elegans *under excess (closed circles), high (open circles) and low food (closed squares) environments. (C) Total fecundity of *C. elegans *under excess, high and low food environments; *n *= 36, 44 and 35 respectively. (D) Growth curves of *C. elegans *under excess (closed circles), high (open circles) and low food (squares) environments, *n *= 49, 38, and 21 respectively. Images show representative adults from high food (upper panel) and low food (lower panel) treatments. Scale bar indicates 100 μm. (E) Hypodermal (hyp7) cell number of young adult *C. elegans *under high and low food environments; *n *= 10 and 9 respectively. (F) Hypodermal (hyp7) ploidy of *C. elegans *(120 h) under excess, high and low food environments; *n *= 19, 254, and 189 respectively. All error bars show 95% confidence intervals, and asterisk show level of significance, *** shows *P *< 0.0001, by ANOVA.

We first set up an experimental system for growing *C. elegans *under DR (also referred as "low food conditions"; see Materials and Methods). As we were not interested in the two adaptive responses of *C. elegans *larvae to DR, i.e. L1 arrest [32] and dauer formation [33], we exposed L3 animals grown in high food (see Materials and Methods) to DR. They produced adults with substantial differences with respect to their longevity (57% longer with DR; Figure 1A), fecundity (67% smaller with DR; Figures 1B and 1C) and body size (63% smaller with DR; Figure 1D).

The reduced fecundity and extended longevity are consistent with previous studies on DR using *C. elegans *grown in liquid media [5]. They are also consistent with *Drosophila*'s experiments where DR induces adults of smaller size [34,4]. However, unlike in *Drosophila*, where the reduction in size is due to a combination of reduced cell number and size, in *C. elegans *there is no alteration in cell number, at least in the hypodermis (Figure 1E), which secretes the cuticle, scales with body size and regulates it through TGF-β signalling [35]. Our data also show that the reduction in body size seen on DR is associated with reduced levels of hypodermal endoreduplication (Figure 1F), which we recently showed drives growth in adult worms [36].

### Food consumption regulates body size but not hypodermal ploidy

How does food level control the endoreduplication and growth of worms? One possibility is that worms monitor the amount of food that they actually eat and adjust their ploidy and growth accordingly. To test this idea we first studied a mutant,*eat-2(ad465)*, that has a defective pharynx and therefore cannot eat properly [37,38]. In effect, *eat-2 *mutants experience constitutive DR. We found that, when grown at high food levels, *eat-2 (ad465) *has a small body size but has wild type ploidy [Tables 1 and 2]. Under DR conditions, *eat-2 (ad465) *behaved like wild-type: its body size was even further reduced and its ploidy decreased by 24% (Table 2). This suggested to us that body size is at least partly controlled by the amount of food that a worm eats, but that hypodermal endoreduplication is not.

**Table 1:** Effect of Dietary Restriction on Body Size. All genotypes show significant (*p *< 0.0001), wild type-like (genotype by environment interaction term; *p *> 0.05), reductions in volume under DR.

	Body size (mm^3^)			
			
Genotype	High Food	Low Food	n	% reduction
N2	0.0051 (± 1 × 10^-4^)	0.0021 (± 1 × 10^-4^)	147, 121	63
*che-2(e1033)*	0.0022 (± 1 × 10^-4^)	0.0009 (± 2 × 10^-4^)	106, 43	62
*che-3(e1124)*	0.0029 (± 2 × 10^-4^)	0.0013 (± 8 × 10^-5^)	19, 12	55
*che-11(e1810)*	0.0027 (± 1 × 10^-4^)	0.0012 (± 1 × 10^-4^)	41, 23	56
*che-13(e1805)*	0.0021 (± 3 × 10^-4^)	0.0009 (± 5 × 10^-5^)	11, 16	57
*osm-5(p813)*	0.0033 (± 2 × 10^-4^)	0.0013 (± 2 × 10^-4^)	26, 20	61
*egl-4(n478)*	0.0063 (± 2 × 10^-4^)	0.0023 (± 1 × 10^-4^)	127, 118	60
*dbl-1(nk3)*	0.0025 (± 1 × 10^-4^)	0.001 (± 6 × 10^-5^)	121, 80	62
*sma-2(e502)*	0.0020 (± 4 × 10^-4^)	0.0010 (± 2 × 10^-4^)	19, 15	50
*sma-3(wk20)*	0.0025 (± 2 × 10^-4^)	0.0010 (± 1 × 10^-4^)	32, 24	60
*sma-4(e729)*	0.0010 (± 1 × 10^-4^)	0.0006 (± 1 × 10^-4^)	33, 22	40
*sma-6(wk7)*	0.0019 (± 3 × 10^-4^)	0.0009 (± 2 × 10^-4^)	39, 22	53
*lon-1(e185)*	0.0050 (± 6 × 10^-4^)	0.0017 (± 3 × 10^-4^)	20, 9	66
*che-2(e1033);dbl-1(nk3)*	0.0019 (± 1 × 10^-4^)	0.0007 (± 4 × 10^-5^)	48, 35	58
*egl4(n478);dbl-1(nk3)*	0.0028 (± 3 × 10^-4^)		9	
*eat-2(ad465)*	0.0020 (± 1 × 10^-4^)	0.0008 (± 4 × 10^-5^)	71, 56	59
*eat-2(ad465);dbl-1(nk3)*	0.0011 (± 1 × 10^-4^)	0.0005 (± 1 × 10^-4^)	85, 45	52

**Table 2:** Effect of Dietary Restriction on Hypodermal Ploidy. All genotypes, unless stated (NS, *p *> 0.05), show highly significant (*p *< 0.0001) alterations from wild type ploidy responses to DR.

	Hypodermal ploidy (xC)			
			
Genotype	High Food	Low Food	n	% reduction
N2	10.9 (± 0.3)	8.4 (± 0.2)	113, 94	23
*che-2(e1033)*	8.6 (± 0.3)	7.5 (± 0.4)	56, 25	13
*che-3(e1124)*	8.4 (± 0.5)	7.5 (± 0.7)	17, 12	11
*che-11(e1810)*	9.2 (± 0.3)	8.5 (± 0.5)	32, 21	8
*che-13(e1805)*	8.4 (± 0.5)	7.5 (± 0.5)	13, 13	11
*osm-5(p813)*	8.8 (± 0.4)	7.6 (± 0.5)	24, 17	14
*egl-4(n478)*	12.3 (± 0.3)	11.6 (± 0.3)	101, 88	5
*dbl-1(nk3)*	7.5 (± 0.5)	6.9 (± 0.4)	51, 32	8
*sma-2(e502)*	7.6 (± 0.7)	7.0 (± 0.5)	14, 11	8
*sma-3(wk20)*	8.2 (± 0.4)	7.0 (± 0.4)	39, 23	15
*sma-4(e729)*	7.4 (± 0.3)	6.4 (± 0.5)	35, 22	14
*sma-6(wk7)*	8.3 (± 0.3)	7.1 (± 0.3)	31, 18	14
*lon-1(e185)*	12.2 (± 0.9)	8.5 (± 1.0)	9, 8	30 NS
*che-2(e1033);dbl-1(nk3)*	9.1 (± 0.5)	7.8 (± 0.5)	29, 18	14
*egl4(n478);dbl-1(nk3)*	8.9 (± 0.8)'		6	
*eat-2(ad465)*	10.1 (± 0.7)	7.7 (± 0.4)	24, 17	24 NS
*eat-2(ad465);dbl-1(nk3)*	8.5 (± 0.4)	7.0 (± 0.4)	43, 23	18

### Endoreduplication requires the sensation of food by ciliated neurons

If the amount of food that a worm actually eats does not control endoreduplication, why do DR worms have low hypodermal ploidies? One possibility is that worms regulate endoreduplication in response to the amount of food that they sense in their environments. Worms sense their environment by means of their amphids, two small sensory organs that are exposed to the environment through pores located near the worm's mouth. Each amphid has 12 neurons from which eight project into the channel that leads to the pore [39-42]. These eight neurons are ciliated and have specialised endings containing receptor proteins that interpret and distinguish between external stimuli [43].

To test whether sensory signals from the amphids are involved in the DR response, we measured body size in various mutants possessing malformed, non-functional, sensory cilia. Consistent with Fujiwara et al. [30], we found that *che-2(e1033) *is smaller than wild type worms under high food conditions (Table 1). This phenotype is shared with all the other sensory cilia mutants examined (Table 1): *che-13(e1805)*, *osm-5(p813), che-3(e1124) *and *che-11(e1810)*. We also investigated whether the sensory mutants become smaller under DR and found that they had wild type responses (Table 1).

Then, to determine if the small body size of the sensory cilia mutants was associated with reduced ploidy we examined the hypodermis of all the sensory mutants. All of these mutants showed a reduction in ploidy (*p *< 0.001) (Table 2). More importantly, when subjected to DR, their ploidy declined only by approximately 11%, compared to a 23% reduction of the wild type (Table 2). We found no significant differences between the hypodermal nuclei number of *che-2(e1033) *and wild type worms (data not shown). These results suggest that signals from the amphids partly control endoreduplication in response to DR.

### EGL-4 mediates the response from sensory cilia

Previous studies have shown that EGL-4, a cGMP-dependent protein kinase, functions downstream of sensory ciliated neurons in wild type worms [30]. Furthermore, mutations in *egl-4 *result in increased body length, altered sensory perception and egg laying behaviour, without affecting cilia structure [44]. To determine whether EGL-4 is required for the regulation of body size and endoreduplication in response to DR, we first characterized the growth of a strong loss-of-function mutant, *egl-4 (n478)*, under normal levels of food. We found these worms to be 21% larger than wild type (Table 1) and possess a 13% higher level of hypodermal endoreduplication (Table 2), while maintaining wild type cell numbers (data not shown).

Surprisingly, under DR, *egl-4 *exhibits a wild type reduction in volume, but importantly, it fails to show a wild type reduction in endoreduplication. Hypodermal polyploidization, in *egl-4 *worms, declines only 5% under DR compared to a 25% decline in N2 (Table 2). Therefore, *egl-4 *defective worms maintain a hyper-endoreduplicated state at their hypodermis even under DR. Their hyper-endoreduplicated state, their failure to show wild type declines in endoreduplication, and the placement of EGL-4 downstream of CHE-2 [30] (also see Table 1 &2), all together suggest that EGL-4 acts as a negative regulator of food dependent endoreduplication.

### DBL-1 signalling regulates the DR endoreduplication response

**Figure 2:**
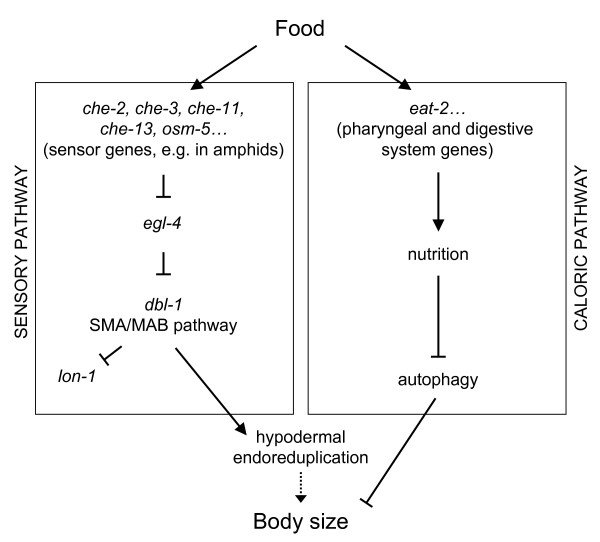
**Model of body size regulation by nutrients availability in *C. elegans *(from L3 onwards)**. Our results suggest that food availability may regulate body size in at least two ways. First, by the "caloric pathway", that is, simply considering that food intake and its absorption by the digestive tract facilitates nutrition, which in turn may inhibit autophagy. Second, by the "sensory pathway", which refers to the sensing food through organs such as the amphids, with their ciliated neurons expressing genes like *che-2*, would inhibit EGL-4. Downstream, this cGMP-dependent protein kinase downregulates DBL-1 signalling, which in turn promotes hypodermal endoreduplication, upregulator of body size [36]. LON-1 inhibition by DBL-1 [28] would not influence ploidy upon nutrient activation. This model explains why the nutrient-dependent regulation that the sensory cilia proteins, EGL-4 and DBL-1 are all playing on hypodermal polyploidization has not been observed for body size; their role on body size, but not upon endoreduplication, may be obscured by the dominant influence of caloric restriction.

DBL-1 is known to be a dose-dependent regulator of body size and endoreduplication in *C. elegans*. This protein activates the SMA-6/DAF-4, Ser/Thr kinase receptor, which in turn is thought to activate the cytoplasmic effectors SMA-2, SMA-3 and SMA-4 [26]. Here we confirm that loss-of-function *dbl-1(nk3*) worms, as well as worms defective for downstream signalling components such as *sma-6*, *sma-2*, *sma-3 *and *sma-4*, all show a 60% reduction in body size when grown in normal food levels (Table 1), similarly to previously reported [45-48]. They also show a ~25% reduction in hypodermal polyploidization (Table 2). To determine the role of DBL-1 in the DR response of wild type worms, all these mutants were subjected to DR and their body size and hypodermal endoreduplication characterized. All mutants showed a marked decrease in size (40% – 60%), responding to DR in a wild type manner (63%; Table 1). More interestingly, when the effects of DR on endoreduplication were examined, mutants deficient for *dbl-1, sma-6, sma-2, sma-3 *and *sma-4 *all show a distinct non-wild type response: endoreduplication declines by approximately 12%, compared to the 23% seen in N2 (Table 2). These results suggest that DBL-1 signalling, as described previously for sensory cilia mutants and *egl-4*, is partially responsible for the regulation of endoreduplication as a response to DR. We note, however, that loss-of-function *lon-1*, placed downstream of the *dbl-1 *pathway [28], behaves as wild type under DR (Table 2). Therefore, we suggest that *lon-1*, despite its role in determining body size and hypodermal endoreduplication, is not part of the polyploidization response to nutrients availability (Figure 2).

### CHE-2 and DBL-1 act in the same pathway to regulate body size and hypodermal ploidy

In order to test the hypothesis that sensory signals and DBL-1 signalling act in the same pathway, we generated double *che-2;dbl-1 *mutants and analysed their size and ploidy levels under standard and DR conditions. When grown in high food conditions, *che-2;dbl-1 *was similar in size and ploidy (*p *> 0.05 for all comparisons), to *dbl-1*, *che-2*, or related genes (e.g. *sma-6*, *che-13*; Tables 1 and 2). The corresponding reduction for both characters under DR was also similar (Tables 1 and 2). This result suggests that *dbl-1 *and the amphid mutants act in the same pathway when controlling body size and hypodermal endoreduplication.

We also asked whether the regulation of body size by food intake per se was affected by DBL-1 signalling. To test this we examined *eat-2;dbl-1 *double mutants. We found that at high food levels, these worms are smaller than either *eat-2 *or *dbl-1 *(Table 1). This additive effect suggests that these genes regulate body size through different pathways, and is consistent with the finding that *eat-2 *worms have normal ploidy.

### EGL-4 negatively regulates DBL-1

To confirm the effect of EGL-4 on the signalling of DBL-1 seen previously [30,31], epistasis analysis was carried out between null mutants *dbl-1 *and *egl-4*. The nature of these mutants allowed a relatively simple analysis because *egl-4 *worms are larger than wild type, whereas *dbl-1 *worms are smaller [45,46] (Table 1). The same thing can be said about hypodermal ploidy (Table 2). Examination of *egl-4;dbl-1 *worms revealed that, though slightly smaller, the double mutant did not significantly differ from *dbl-1 *worms in either adult volume or hypodermal ploidy (*P *> 0.05, for body size and ploidy; Tables 1 and 2, respectively), but it did with respect to *egl-4 *worms (*P *< 0.0001, for body size and ploidy).

## Discussion

Growth is a fundamental part of biology, yet its regulation is still poorly understood [12]. The notion that growth responds passively to nutrient availability has been replaced with the idea that growth is actively regulated in response to constant monitoring of nutrient availability in the external environment. We observed that when *C. elegans *is exposed to a low food environment there is a reduction in adult body size, similar to the reductions seen in other organisms e.g. *Drosophila *and *Daphnia *[4,34,49,50]. However, in contrast to these organisms, the stunting in *C. elegans *is not due to a lack of cell proliferation, which implies that it is due to a reduction in cell size.

In order to investigate how DR controls adult body size in *C. elegans*, we studied the growth of wild type and mutant worms subjected to high and low food regimes. We found that all of our mutants became smaller by about the same amount (60%) at low food levels. This absence of interaction between food and genotype on growth might mean that none of the genes examined are involved in the dietary-dependent regulation of growth, but it could also simply mean that severe DR has additional effects.

For this reason we needed a more subtle way of examining the effects of DR on worm development. We have previously shown that hypodermal endoreduplication is required for growth in adult *C. elegans *[36]. Strikingly, we also found that DR inhibits hypodermal endoreduplication and so adult ploidy. This result gave us a sensitive assay for the effects of DR on the worm's development. We found that mutations in several genes mimic the DR response: even at high food levels, mutations that disrupt sensory or DBL-1 signalling show reduced ploidy and body size. That suggested to us that these genes might be involved in the DR endoreuplication response. This inference was confirmed when we examined these mutants under DR: in each case, the reduction in ploidy normally found at low food levels was largely abrogated. An even more striking lack of response to DR was also found in a large mutant that disrupts *egl-4*, a cGMP-dependent protein kinase previously associated with food sensing and food dependent behaviour [30].

These results, and our epistasis experiments, suggest a model in which the amphids monitor nutrient availability and activate a downstream signalling pathway involving the growth repressor EGL-4 (Figure 2). This kinase in turn regulates the DBL-1/SMA/MAB pathway, which positively regulates hypodermal endoreduplication. As the observed body size reduction under DR for both the sensory and DBL-1 signalling pathway mutants was similar to that of wild type (Table 1), we suggest that the main effects of DR on body size do not arise from the lack of endoreduplication, but rather from some other unknown pathway. A likely candidate could be what we call the "caloric pathway" in Figure 2. That is, the severe food restriction under DR could be masking the "sensory pathway" on body size when this one is impaired (e.g. in *dbl-1(nk3)*; Figure 2). Reduction in food may prevent DBL-1 like mutants, whose endoreduplication levels do not drop as much as wild type under DR, from growing larger. Nevertheless, the reduced ploidy programmed by the sensing of lower levels of food (Table 2) must contribute to the stunting, since previous work shows a cause-and-effect relationship between endoreduplication and adult growth [36]. Consistent with our model, we showed that *eat-2 *mutants, one of the genes active in the feeding mechanism, has small size but normal ploidy, and that it reduces both characters in a wild type manner under DR (Tables 1 and 2). Recent work suggests that *eat *mutants have small body sizes due to increased autophagy [38], which is also included in our model (Figure 2).

## Conclusion

How do our results relate to other animal models? Endoreduplication in *Drosophila *depends on a mitogen from the fat body that is regulated in a nutrition-dependent manner [51], which may suggest at least an underlying common plan beyond their differences (see Introduction)[52]. However, one of the proteins studied here, EGL-4, is a key regulator of nutrient responses not only in worms but, with the generic name of cGMP-dependent protein kinase, in organisms such as honeybees and fruit-flies controlling their foraging behaviour [53]. It is somewhat surprising that loss-of-function *egl-4 *has a change in hypodermal endoreduplication in high vs. low food which is half of what it is observed for the sensory ciliated or for the *dbl-1*-related mutants (Table 2). We think that this difference can be explained because *egl-4*'s role in nutrient-dependent growth may be central, not shared with other proteins in parallel positions, whereas the various sensor genes investigated may be acting in parallel, either among themselves, or in relation to other genes or pathways (similarly for DBL-1 and the SMA/MAB pathway). In agreement with this, *egl-4 *is considered a highly pleiotropic gene, a main regulatory hub, not only mediating body size but longevity, locomotion feeding, and other processes [54].

## Methods

### Strains

Apart from the wild type strain N2, the following mutant strains were used, which were obtained from the *Caenorhabditis elegans *Genetics Center. Mutations are listed by linkage group: LGI: *che-3(e1124), che-13(e1805)*; LGII: *sma-6(wk7)*, *eat-2(ad465)*; LGIII: *lon-1(e185), sma-2(e502), sma-3(wk20), sma-4(e729)*; LGIV: *egl-4(n478)*; LGV: *che-11(e1810), dbl-1(nk3)*; LGX: *che-2(e1033), osm-5(p813)*. We also used double mutants that we produced through crosses of the previous strains. Double mutants *eat-2(ad465);dbl-1(nk3) *were confirmed by PCR and sequencing using the following primers: 5'-eat-2: 5' TGATCACCCTAGTTGTCTGG; 3'-eat-2: 5' AGTGTAGAGGTACTGTATGG; 5'-dbl-1: 5' CATGGACAAACATCGGGGA; and 3'-dbl-1: 5' CGTGTACACAAATCTGTTCG. *che-2(e1033);dbl-1(nk3) *was generated by crossing heterozygous *dbl-1(nk3) *males with *che-2(e1033) *hermaphrodites. Then, their F1 progeny was PCR-screened for the *nk3 *allele, and double mutants in F2 were confirmed by PCR for both *nk3 *and *e1033 *alleles, and by DNA sequencing with oligonucleotides 5'- dbl-1, 3'- dbl-1, 5'-che-2: 5' AGATGGATGTTTACTGCC, and 3'-che-2: 5' GAGAATGACACAATGTGG.

All strains and experiments were maintained at 20°C.

### Dietary restriction

We developed a novel method of dietary restriction (DR) on solid media. Three different food treatments are described within this study: excess, high, and low food treatment plates. Excess food plates: 100 μl of 5.19 × 10^8^/ml *E. coli *(OP50)-Luria broth was spread around the centre of 5.5 cm NGM plates and left at room temperature for 24 hours before being killed by exposure to UV light for 1 hour. High food plates were prepared as excess food plates, but were exposed to UV light for 1 hour immediately after preparation. Low food plates were prepared as high food plates, but using a suspension of 3.95 × 10^7^/ml *E. coli *(OP50)-Luria broth. For each experiment, the same *E. coli *culture was used for each food treatment. Treatment plates were replaced every 24 h during worm growth experiments to prevent depletion food source.

### Body size analysis

Growth curves were determined for each strain, from worms grown individually on 5 cm Petri dishes. At 24 h intervals from 36 h to 120 h post hatching, images were captured using a video camera (JVC KY-F50) attached to a dissecting microscope (×50), and analyzed with OBJECT-IMAGE 1.62. Length and area were measured from pictures of individual worms and calibrated from a 1 mm graticule. Volume was calculated assuming cylindrical body shape using the formula (pi*length*(area/length)^2^/4) [36,46]. All comparisons of body size use Log-transformed data.

### Hypodermal ploidy analysis

Upon completing growth (120 h), worms were fixed in Carnoy's solution for 24 h, stained in a 0.007 mg/ml solution of 4',6-diamidino-2-phenylindole dihydrochloride (DAPI) [36,48,55,56] and viewed under a Leitz epifluorescence microscope. Images of hypodermal and ventral cord nuclei were collected using a CV-M300 video camera, and analyzed using OBJECT-IMAGE 1.62. C values of hypodermal nuclei were estimated by dividing their DAPI-based densitometric quantifications by an average of those values from ventral cord nuclei (divided by two) in the same microscopic preparations [36].

### Cell number analysis

Young adult worms were anesthetized with 0.1 M sodium azide [57], and viewed at ×1000 under differential interference contrast optics with a Nikon Eclipse E600 microscope. All nuclei, excluding neuronal and seam cells, between the posterior pharyngeal bulb and anus were counted. Images were captured with a CV-M300 camera and reconstructed by using Adobe PHOTOSHOP 4.0.

### Longevity analysis

We analysed Kaplan-Meier survival distributions, which are based on a discrete stepped survival curve, adding time specific data as each death occurs. Individuals that died from internal hatching of eggs (bagging), or crawled off the plate were censored. Censoring allows the inclusion of individuals that were lost to the study, and thus contribute towards knowledge of survivorship, but nothing to the knowledge of age at death. Log-rank tests were performed to determine if survival curves were significantly different from each other.

### Egg-laying assays

Individual worms were placed OP50-seeded 5.5 cm NGM plates before adult moult occurred and transferred to a fresh plate every 24 h. Total fecundity was measured with only fertilized eggs and larvae being included in the count.

### Statistical analyses

Data analysis was undertaken using JMP 3.2 (SAS Institute, Cary NC, USA). Body size and ploidy data were compared across food level and genotype using a standard two-way ANOVA, including a genotype by environment interaction term, to determine responses of each genotype to DR. A food level by genotype interaction term allowed the comparison of each mutant genotype's response to DR to that of wild type. Ratios, between high and low food groups, were not used in this analysis.

## Authors' contributions

LST designed the study, created the dietary restriction methodology, performed body size, ploidy, cell number, longevity, egg laying and statistical analysis. EL performed body size and ploidy analysis. AGS did ploidy analysis. AML conceived the study and participated in its design. The paper was written by LST and AGS, with the help of AML and EL.
